# The molecular detection of carbapenem markers with a two-levels amplification screening protocol: epidemiological and resistome insights

**DOI:** 10.3389/fmicb.2024.1346442

**Published:** 2024-02-23

**Authors:** Maddalena Calvo, Giuseppe Migliorisi, Gaetano Maugeri, Dafne Bongiorno, Carmelo Bonomo, Emanuele Nicitra, Guido Scalia, Stefania Stefani

**Affiliations:** ^1^U.O.C. Laboratory Analysis Unit, A.O.U. “Policlinico-San Marco”, Catania, Italy; ^2^Department of Biomedical and Biotechnological Sciences (BIOMETEC), University of Catania, Catania, Italy

**Keywords:** carbapenem-resistance, infection control measures, molecular assays, resistome analysis, screening protocol

## Abstract

**Objectives:**

Carbapenem-resistance is a challenging healthcare concern and require specific stewardship programs. Monitoring workflows include the identification from surveillance samples, such as rectal swabs. Although culture assays represent the gold standard, data report a significant effectiveness in detecting carbapenemases genes directly from rectal swabs. The aim of this study was to evaluate the REALQUALITY Carba-Screen kit (AB ANALITICA, Padova, Italy) in detecting carbapenemases genes directly from rectal swabs, also comparing its effectiveness to culture assays results. A next-generation sequencing (NGS) was performed to investigate the positive samples about resistance markers and sequence type (ST).

**Methods:**

A number of 136 rectal swabs were collected from the University Hospital Policlinico of Catania critical wards. The samples simultaneously underwent culture and molecular assays (REALQUALITY Carba-Screen kit). The molecular method included two-steps. The first step (1 h and 6 min) rapidly excluded negative samples, while the second one (1 h and 6 min) included only positive samples for a resistance confirmation. All the positive culture samples underwent NGS analysis.

**Results:**

Statistical evaluations demonstrated high sensitivity (100%) and detection rates (92.6%) for the REALQUALITY Carba-Screen kit, which mostly correlated to the standard workflow. All the culture positive results matched the positive molecular results, which were mainly confirmed by the NGS resistome analysis. The identified ST appeared to be diversified and different from the clinically significative strains of the same setting, furnishing interesting epidemiological evidence.

**Conclusion:**

The molecular detection allowed a coordinate approach in a high-prevalence multi-drug-resistance area. The rapid identification with a multi-step procedure accelerated the infection control procedures, while the preliminary negative results reduced the overtreatment episodes. The molecular method efficacy was confirmed through the NGS. In conclusion, the molecular screening could initially lead to a more conservative approach, which may be reevaluated after a culture result about the microorganisms’ identification and susceptibility profile.

## 1 Introduction

Carbapenem-resistance prevention is one of the most challenging concerns in healthcare settings, which need to organize stewardship programs and isolation precautions to control these alert microorganisms’ spread (Caliskan-Aydogan and Alocilja, 2023). The European surveillance networks highlight the importance of carefully monitoring carbapenem-resistant *Enterobacterales* (CRE) and *Acinetobacter baumannii*, which hardly respond to common antimicrobial therapies and express a massive endemic diffusion capability among hospital settings (CDC, 2019). Literary data often describe the clonal diffusion of identical clones among the same extended health-care setting (CDC, 2019; Ochońska et al., 2021). Italy recently recorded a carbapenem-resistant *K. pneumoniae* rate of 26.7% and a carbapenem-resistant *E. coli* percentage of 23.8%. Finally, *Acinetobacter* spp. reached 86.9% in the same country (EARS-Net, 2023). These data suggest the importance of performing a precise surveillance protocol in the Italian hospital setting. Specifically, Sicily accounted for 51.5% of carbapenem-resistant *K. pneumoniae*, 1.9% of carbapenem-resistant *E. coli*, and 88.4% of carbapenem-resistant *A. baumannii* (Regione siciliana, 2019). In addition, specific surveillance protocols about colistin-resistant strains have recently been planned within European countries to monitor both carbapenem-resistant *Enterobacterales* and *Acinetobacter* spp. (Lötsch et al., 2020). Carbapenem-resistant microorganisms may be long-term persistent colonizing agents, thus carbapenem-resistance screening programs need to be settled since patients’ admission. Rectal swabs represent an ideal surveillance specimen due to the possibility of the gastrointestinal tract being a reservoir for carbapenem-resistant microorganisms (Tacconelli et al., 2014; Kim et al., 2018). Culture assay still represents the most reliable method in determining a possible carbapenem resistance on grown colonies from rectal swabs (Adler et al., 2011; Foschi et al., 2020). This conventional workflow includes antimicrobial susceptibility testing (AST) to provide MIC values for carbapenems. Despite the high specificity and reproducibility rates, culture methods require a long turn-around time (TAT), which is not optimal for managing critical patient courting and isolation. Data report a significant effectiveness in detecting carbapenemase genes directly from rectal swabs. Direct detection leads to a TAT reduction and helps critical settings manage colonized patients (Shorten et al., 2016; Del Bianco et al., 2019; Cury et al., 2020; Alqahtani et al., 2021; García-Fernández et al., 2022; EUCAST, 2023).

The aim of this prospective experimental study was to compare the REALQUALITY Carba-Screen kit (AB ANALITICA, Padova, Italy) to standard culture-based methods in detecting carbapenemases genes directly from rectal swabs. The evaluation aimed to demonstrate the reliability of a fast diagnostic workflow for screening protocols, allowing rapid patient courting and alert for fast control purposes. Furthermore, the protocol applied a next-generation sequencing for the grown colonies related to all the REALQUALITY Carba-Screen positive specimens. This ultimate step allowed to evaluate circulating MDR clones in stool samples and confirm molecular results already obtained directly from clinical samples.

## 2 Materials and methods

**GRAPH 1:**
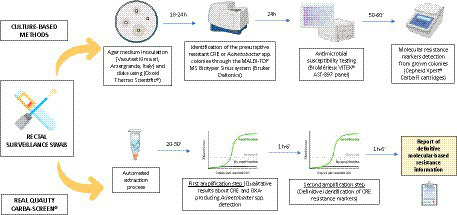
Graphical representation of the applied experimental workflow. Each collected surveillance swab was processed simultaneously through culture-based and molecular methods. The graph was created through the Biorender.com database.

The experimental protocol was performed at the University Hospital Policlinico of Catania during a one-month evaluation (April 2023–May 2023), where routinary surveillance rectal swabs integrate a periodical collection as a prevention. A total of 136 rectal swabs derived from Hematology, Intensive Care, and emergency units. All these samples represented a part of conventional antimicrobial resistance surveillance programs. These programs specifically include a rectal screening for CRE microorganisms on patients’ admission and weekly during their recovery period. All the samples were collected using ESwab, a screw-cap tube filled with 1 mL of Liquid Amies Medium, and a regular FLOQSwabs^®^ (COPAN Italia SpA., Brescia, Italy). Rectal swabs were transported to the Laboratory Analysis Unit and processed within 24 h. Specifically, they simultaneously went through the traditional culture-based method and the experimental molecular workflow with the REALQUALITY Carba-Screen^®^ kit (AB ANALITICA, Padova, Italy). On that premise, the study did not involve supplementary samples or direct intervention on patients and only included laboratory processes. Data anonymization protected all the patients whose informed consent was not mandatory for the local Research Ethics Committee. Only residual leftover specimens from the eSwabs sample experienced the molecular technique. Finally, the protocol included next-generation sequencing (NGS) to confirm molecular results and enrich epidemiological evaluations of all the culture-positive specimens. Graph 1 describes all the stages of the experimental workflow.

### 2.1 Culture-based methods and sample storage

A 10 μL aliquot of liquid rectal swabs transport medium was inoculated into a MacConkey agar plate (Vacutest Kima srl, Arzergrande, Italy) using a 10 μg meropenem disk (Oxoid Thermo Scientific^®^) as an indicator for possible carbapenemases production. The plates faced a 37°C overnight incubation period, and the grown bacteria endured identification protocols.

The eventual meropenem inhibition diameter [European Committee on Antimicrobial Susceptibility Testing (EUCAST)] (3dmedcare, 2015; EUCAST, 2017) allowed the presumptive identification of Enterobacterales or Acinetobacter spp. The MALDI-TOF MS Biotyper Sirius system (Bruker Daltonics) identified the carbapenem-resistant strains and the BioMérieux VITEK^®^ AST-397 panel provide antimicrobial susceptibility testing. The MIC values were analyzed according to an EUCAST expert evaluation (3dmedcare, 2015; EUCAST, 2017). The meropenem, meropenem/vaborbactam, ceftazidime/avibactam, ceftolozano/tazobactam and colistin MIC values were confirmed through a broth microdilution (BMD). The Kirby-Bauer method determined the cefiderocol MIC values. The phenotypically resistant strains grown colonies faced a supplementary molecular process to detect KPC, OXA48, NDM, VIM, or IMP genes, requiring 50–60 min by Cepheid Xpert^®^ Carba-R cartridges.

The entire process required a minimum of 48 h to provide a definitive result. All the grown carbapenem-resistant Enterobacterales and Acinetobacter spp. colonies were stored in 20% glycerol and brain heart infusion (BHI) vials at −80°C.

### 2.2 Real quality Carba-Screen experimental protocol

Each sample followed the scheme summarized in Graph 1.

A 200 μL quantity after a vortex step and a mixing with 20 μL of K proteinase, was extracted with a semi-automated nucleic acid extraction system (ANDiS 360 Automated Nucleic Acid Extraction System, 3D Medicines Biomedical Technologies), which allowed the simultaneous processing of 16 samples. The extraction process followed the manufacturer’s instructions (ANALITICA, 2022). Briefly, from an aliquot of 60 μL DNA elution, 5 μL was mixed with 20 μL of Master Mix. Then, the AriaDx system (Agilent Technologies) performed a qRT-PCR. The REALQUALITY Carba-Screen kit (AB ANALITICA, Padova, Italy) was validated both on rectal swabs and bacterial colonies. In our specific investigations, the kit was only used directly on rectal swab material. The workflow involved a two-step amplification procedure. The first part was considered as a screening using the REALQUALITY Carba-Screen Real Time mix, which is able to distinguish negative samples from class B, A, and D carbapenemases and/or *Acinetobacter* spp. OXA positive samples. Specifically, the technology can detect OXA-23 like, OXA-24 like, OXA-51 like with promoter ISAba1 and OXA-58 like.

All the positive samples endured a second differential step, which involves Mix Carba-B (for bla_NDM_, bla_VIM_, and bla_IMP_) and Mix Carba a + D (for bla_KPC_, bla_OXA48_, and *mcr1,2,4*). Each master Mix contains an internal control (IC) based On The amplification of The bacterial 16S RNA gene, which ensures The sample adequacy To amplification processes. The product also includes a dUTP/UNG system To prevent contaminations. The amplification processes were used according To The manufacturer’s instructions, through The Aria dx software (Agilent Technologies) (Bongiorno et al., 2023; QPCR, 2023).

### 2.3 Next-generation sequencing

A whole genome sequencing (WGS) analysis involved all the grown colonies from positive Carba-Screen samples. The WGS analysis aimed to define the Sequence Type (ST), confirm the carbapenem-resistance through a resistome analysis, and identify the virulome of all the isolates. The QIAGEN QIAamp^®^ DNA Mini Kit (Ref. 51304, QIAGEN, 40724 Hilden, Germany) allowed the colony extraction. The Eppendorf BioPhotometer^®^ D30 and the fluorimeter Qubit dsDNA BR Assay Kit, respectively, evaluated purity and quantity of the initial sample, favoring the DNA quantification (Ref. 32850, Invitrogen, 92008 Carlsbad, CA, United States) (FASTQ, 2021).

The Molecular Biology Laboratory from the University of Catania performed the NGS sequencing, placing 100 ng of each sample on an Illumina MiSeq platform according to the manufacturer’s instructions provided in Watchmaker DNA Library Prep kit with Fragmentation–Watchmaker Genomics^®^ (Ref. 7 K0013-024, 5744 Central Avenue, Suite 100 Boulder, CO 80301, United States). Indexes were provided with Twist Universal Adapter System (16 Indexes, 16 Samples) (Ref. 101307, Twist Bioscience, HQ 681 Gateway Blvd, South San Francisco, CA 94080FAQ).

The fluorometric Qubit dsDNA HS Assay Kit (Ref. Q32851, Invitrogen, Carlsbad, CA 92008, United States) and the Agilent^®^ High Sensitivity DNA Kit (Ref. 5067-4626) allowed the libraries quantification and quality evaluation. Denature and dilute libraries were performed following the “Denature and Dilute Libraries Guide” protocol provided by Illumina^®^, choosing 8.5 pM as the loading concentration. Finally, sequencing was performed using the MiSeq Reagent Kits v3 (Ref. 15043895, Illumina, Inc., 92122, San Diego, CA, United States). The Sample Sheet was created using the Local Run Manager v3 software, and following the instructions in the Local Run Manager v3 Software Guide provided by Illumina (Martin, 2011; FASTQ, 2021).

The protocol was completed using two different types of analysis with the QIAGEN CLC Genomics Workbench software, following the User Manual for CLC Microbial Genomics Module v22.0, released on January 4, 2022 (QIAGEN, Aarhus, 8000 Denmark). The software assigned resistance, virulence, and MLST genes (FASTQ, 2021). The bioinformatic analysis was also manually performed using further tools. Specifically, TrimGalore (v0.5.0) (Wick et al., 2017; Krueger, 2022) removed the adapter sequence, while *de novo* bacterial sequence assembly was executed through Unycler (v0.4.8) (Lam et al., 2021) with the Illumina-only assembly modality. The identification of known virulence factors, resistance genes, and capsule loci was performed using Kleborate (v2.2.0), and the Kaptive (Seemann, 2014; Wyres et al., 2016) command for *Klebsiella pneumoniae* instead of *A. baumannii* BacPipe. Prokka (v1.13) (Danecek et al., 2021) was used for bacterial annotation. They aligned the output assemblies of Unycicler with several through *bwa* (0.7.17) to identify punctual mutations in selected genes, while BFTftools (1.3.1) (Li, 2011; Quijada et al., 2019) provided the variants extraction. Specifically, *lamB* (ARO:3007420), ompk35 (ARO:3003966), ompk36 (ARO:3003968) and ompk37 (ARO:3004122) were used as references.^1^ Metagenome assemblies were also screened for antimicrobial resistance genes using TORMES (version 1.3.0) (Dhanya et al., 2022). All the commercial kits and interpretation software were applied following the manufacturer’s instructions.

### 2.4 Statistical evaluation and comparisons

All the gathered data experienced a statistical evaluation of agreement rates between conventional and molecular methods. The statistical analysis regarded only valid samples obtained through the application of the REALQUALITY Carba-Screen technique. Specificity and sensitivity rates together with positive and negative predictive values were calculated. A comparison of the two different turn-around time has been also provided. The REALQUALITY Carba-Screen results were also compared to the WGS confirmation and the phenotypical susceptibility profiles of the analyzed strains.

## 3 Results

A total of 29 (21.3%) samples tested positive after the application of the culture-based methods, which otherwise revealed 107 (78.7%) negative results. Table 1 summarizes these percentages.

**Table 1:** Culture-based methods and Carba Screen^®^ assay gathered results.

Samples	Culture exams	Carba Screen assay
Tested samples	136	136
Valid samples	136 (100%)	126 (92.6%)
Invalid samples	0	10 (7.4%)
Positive valid samples	29 (21.3%)	52 (41.3%)
Negative valid samples	107 (78.7%)	74 (58.7%)

Among the 136 tested samples, 126 (92.6%) got a valid REALQUALITY Carba-Screen result, meaning that internal controls and amplification processes were correctly completed. Otherwise, 10 (7.4%) samples resulted in invalid processes and did not enter into the statistical evaluation. A number of 52 (41.3%) valid samples resulted as positive for carbapenem-resistant *Enterobacterales* or *Acinetobacter* spp. On the other hand, 74 (58.7%) valid samples tested negative for the same targets. A global detection rate of 92.6% was obtained. Table 1 summarizes the REALQUALITY Carba-Screen gathered results, while Table 2 includes the statistical evaluations of the performance.

**Table 2:** Statistical evaluation about the conventional methods and the Carba-Screen results comparison.

Statistical evaluations	
**Sensitivity** (culture positive samples which also tested positive to Carba-Screen techniques)	100%
**Specificity** (culture negative samples which also tested negative to Carba-Screen Techniques)	76.3%
**Positive predictive value** (positive REALQUALITY Carba-Screen kit samples which also tested positive to culture methods)	55.8%
**Negative predictive value** (negative REALQUALITY Carba-Screen kit samples which also tested negative to culture methods)	100%
**Agreement** (global percentage indicating the number of REALQUALITY Carba-Screen kit result which matched the culture methods results)	81.7%
**Accuracy** (capability to match all the true positive among the involved samples)	100%

The invalid samples evaded the statistical evaluations. The agreement to the culture-based methods was calculated based on the number of Carba-Screen tests which perfectly matched the culture assays. Consequently, all the positive Carba-Screen results which tested negative after the culture assay were considered as discrepancies. The accuracy percentage accounted for all the true positive samples, also including culture negative results which tested positive after the application of a conventional molecular method (Cepheid GeneXpert Carba-R) and the REALQUALITY Carba-Screen Kit.

The analysis revealed an agreement of 81.7% between the molecular assay and the culture-based methods. Specifically, all (100%) of the culture-positive samples (29) also tested positive through the molecular assay, while 74 (76.3%) of negative samples confirmed the same result with the REALQUALITY Carba-Screen kit. Among the culture-negative samples, 23 (23.7%) results tested positive after the application of the molecular assay due to the higher consolidated sensitivity rate of the Real-Time PCR protocol.

The conventional Cepheid GeneXpert Carba-R cartridge confirmed the REALQUALITY Carba-Screen kit result on this particular subset. Moreover, the molecular assay revealed a sensitivity rate and a negative predictive value of 100%. However, the specificity rate (76.3%) and the positive predictive value (55.8%) were lower than the above-mentioned percentages.

The molecular positive samples revealed significant percentages of KPC and NDM-producing *Enterobacterales* together with OXA-producing *Acinetobacter*. Remarkably, OXA48- or *mcr*-producing *Enterobacterales* did not result during the time-period of our experimental study. Table 3 indicates all the details about Carba-Screen’s identified targets among all the tested samples.

**Table 3:** Details about Carba-Screen identified targets among all the tested samples.

Detected genes (positive valid samples)	Samples number
*Enterobacterales* KPC	10 (19.2%)
*Enterobacterales* OXA-48	0
*Enterobacterales* mcr-1,2,3,4	0
*Enterobacterales* NDM	6 (11.5%)
*Enterobacterales* VIM	2 (3.8%)
*Enterobacterales* KPC + NDM	6 (11.5%)
*Enterobacterales* KPC + VIM	2 (3.8%)
*Acinetobacter* spp. OXA	6 (11.5%)
*Acinetobacter* OXA + *Enterobacterales* KPC	4 (7.7%)
*Acinetobacter* spp. OXA + *Enterobacterales* KPC + *Enterobacterales* NDM	1 (1.92%)
*Acinetobacter* spp. OXA + *Enterobacterales* NDM	12 (23.1%)
*Acinetobacter* spp. OXA + *Enterobacterales* NDM + *Enterobacterales* VIM	3 (5.8%)

A turn-around time (TAT) analysis has been provided. The conventional culture-based method requires a minimum of 18–24 h to obtain a species identification and a presumptive meropenem resistance, prolonging this interval to 48 h to gather a definitive susceptibility report and information about a precise resistance marker. Otherwise, the REALQUALITY Carba-Screen method allowed us to articulate a two-step process after a 20–30-min extraction phase.

**GRAPH 2:**
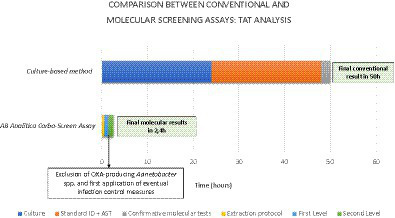
TAT differences between the gold standard method and innovative molecular techniques in detecting resistance markers.

Specifically, the first level revealed a qualitative and preliminary result about positive or negative samples requiring 1 h and 6 min, including also information about the eventual presence of OXA-producing *Acinetobacter baumannii* allowing the activation of infection control procedures. All the positive samples underwent the second level, which allowed us to identify the specific resistance marker, prolonging the process for a further 1 h and 6 min. Globally, the molecular workflow demanded about 160 min. Graph 2 shows a TAT comparison between the culture-based method and the Carba-Screen molecular assay.

All the samples which tested positive for the culture method related to positive rectal samples on the Carba-Screen molecular assay went through a resistome analysis by using Next generation sequencing (NGS). Specifically, 25 *Klebsiella pneumoniae* and 4 *Acinetobacter baumannii* emerged from the assays. *K. pneumoniae* strains belonged to several sequence type (ST). Notably, the analysis registered ST395 (n. 10), ST101 (n. 4), ST17 (n. 5), ST307 (n. 3), ST46 (n. 2), and ST35 (n. 1). All the *K. pneumoniae* ST were closely related to specific capsular types. A number of 18 cases (72%) revealed a complete match between WGS analysis and Carba-Screen results about carbapenemase genes. Otherwise, 7 *K. pneumoniae* strains (28%) showed discrepancies between the two analyses. Specifically, 4 cases described NDM and KPC genes detection after the Carba-Screen application, while the resistome analysis only reported NDM amplification. Furthermore, two strains showed a KPC and NDM Carba-Screen amplification, while the NGS exclusively informed on the KPC presence.

Finally, 1 case reported a KPC and VIM genes Carba-Screen detection, but the resistome analysis did not reveal carbapenemase genes. The same case was confirmed to carry the OXA-9 gene together with ompK37 alterations. Regarding further resistance information, the resistome analysis highlighted the eventual presence of mutations in *lamb* genes (encoding for a maltose-inducible membrane protein) and *ompK* genes (encoding for outer membrane proteins) for all the *K. pneumoniae* genes. *A. baumannii* strains belonged to the sequence type 2 (ST2). In these strains, Carba-Screen totally matched (100%) the resistome analysis, which revealed the presence of OXA-23 (100%) and OXA-66 (50%) beyond the presence of the constitutive OXA-51. In conclusion, no carbapenem-resistant isolates were missed by the tested system.

Table 4 shows the colistin and β-lactams susceptibility profiles of *K. pneumoniae* and *A. baumannii* strains to support the investigated evidence. Additionally, Tables 5, 6 summarize the comparison between the Carba-Screen and the NGS results about the positive *K. pneumoniae* and *A. baumannii* samples.

**Table 4:** Phenotypical colistin and β-lactam susceptibility profile of *K. pneumoniae* (Kp) and *A. baumannii* (Ab) strains.

	Strain	Meropenem MIC	Meropenem/vaborbactam MIC (mg/L)	Ceftazidime/avibactam MIC (mg/L)	Ceftolozano/tazobactam MIC (mg/L)	Cefiderocol Kirby-Bauer result (mm)	Colistin MIC (mg/L)
ST395	Kp 229	16	>256	>256	16	22 mm	0.5
ST395	Kp 322	32	>256	>256	16	24 mm	0.5
ST395	Kp 326	32	>256	>256	16	22 mm	0.5
ST395	Kp 233	32	>256	>256	16	22 mm	0.5
ST395	Kp 236	16	>256	>256	16	20 mm	0.5
ST395	Kp 273	32	>256	16	8	22 mm	0.5
ST395	Kp 304	64	>256	16	8	22 mm	0.5
ST395	Kp 281	32	>256	16	16	20 mm	0.5
ST395	Kp 267	16	>256	64	8	20 mm	0.5
ST395	Kp 300	64	>256	64	8	20 mm	0.5
ST17	Kp 234	16	1	0.5	8	20 mm	0.5
ST17	Kp 241	16	2	0.5	16	20 mm	0.5
ST17	Kp 325	16	0.064	0.5	8	22 mm	0.5
ST17	Kp 324	16	1	0.5	8	20 mm	0.5
ST17	Kp 323	32	0.064	0.5	8	22 mm	0.5
ST101	Kp 209	16	2	1	16	18 mm	0.5
ST101	Kp 244	16	2	1	16	20 mm	0.5
ST101	Kp 230	64	1	1	16	18 mm	0.5
ST101	Kp 312	64	0.064	0.5	8	20 mm	0.5
ST307	Kp 316	16	2	1	1	22 mm	0.5
ST307	Kp 341	16	1	1	16	19 mm	0.5
ST307	Kp 285	16	2	2	16	20 mm	0.5
ST46	Kp 327	16	0.064	0.5	8	20 mm	0.5
ST46	Kp 248	16	1	0.5	8	19 mm	8
ST35	Kp 232	16	>256	>256	16	22 mm	0.5
ST2	Ab 292	16	NA	NA	NA	17 mm	0.5
ST2	Ab 230/2	128	NA	NA	NA	20 mm	0.5
ST2	Ab 250	16	NA	NA	NA	22 mm	0.5
ST2	Ab 311	64	NA	NA	NA	18 mm	0.5

NA, Not applicable according to EUCAST guidelines.

**Table 5:** Comparison between Carba-Screen results and NGS resistome analysis related to *K. pneumoniae* (Kp) isolates.

						Realquality carbascreen screening (1° level)			Realquality carbascreen identification (2° level)						NGS (resistome analysis related to β-lactams resistance)												
						Class B	Class A + D	*Acinetobacter* OXA	Carba B			Carba A + D															
Strain	Carbapenem-resistant phenotype	Identification	MLST	k-locus	o-locus				NDM	VIM	IMP	KPC	OXA-48	*mcr*	bla_NDM_	bla_KPC_	bla_SHV_				bla_CTX_	bla_TEM_		bla_OXA_		*LamB* mutants	ompK35/36/37 mutants
Kp 229	Positive	*K. pneumoniae*	ST395	2	O1/O2v1	Positive	Positive	Negative	bla_NDM_	Negative	Negative	bla_KPC_	Negative	Negative	*NDM-1*				*SHV-187*		*CTX-M-15*			*OXA-1*		*Yes*	Yes (ompK36, ompK37)
Kp 322	Positive	*K. pneumoniae*	ST395	2	O1/O2v1	Positive	Positive	Negative	bla_NDM_	Negative	Negative	bla_KPC_	Negative	Negative	*NDM-1*				*SHV-187*		*CTX-M-15*			*OXA-1*		*Yes*	Yes (ompK36, ompK37)
Kp 326	Positive	*K. pneumoniae*	ST395	2	O1/O2v1	Positive	Positive	Negative	bla_NDM_	Negative	Negative	bla_KPC_	Negative	Negative	*NDM-1*		*SHV-61*	*SHV-152*			*CTX-M-15*			*OXA-1*		*Yes*	Yes (ompK36, ompK37)
Kp 233	Positive	*K. pneumoniae*	ST395	2	O1/O2v1	Positive	Negative	Negative	bla_NDM_	Negative	Negative	Negative	Negative	Negative	*NDM-1*				*SHV-187*		*CTX-M-15*			*OXA-1*		*Yes*	Yes (ompK36, ompK37)
Kp 236	Positive	*K. pneumoniae*	ST395	2	O1/O2v1	Positive	Negative	Negative	bla_NDM_	Negative	Negative	Negative	Negative	Negative	*NDM-1*				*SHV-187*		*CTX-M-15*			*OXA-1*		*Yes*	Yes (ompK36, ompK37)
Kp 273	Positive	*K. pneumoniae*	ST395	2	O1/O2v1	Positive	Negative	Positive	bla_NDM_	Negative	Negative	Negative	Negative	Negative	*NDM-1*						*CTX-M-15*	*TEM-181*		*OXA-1*		No	No
Kp 304	Positive	*K. pneumoniae*	ST395	2	O1/O2v1	Positive	Negative	Positive	bla_NDM_	Negative	Negative	Negative	Negative	Negative	*NDM-1*						*CTX-M-15*		*TEM-122*	*OXA-1*		*No*	Yes (ompK36)
Kp 281	Positive	*K. pneumoniae*	ST395	2	O1/O2v1	Positive	Negative	Positive	bla_NDM_	Negative	Negative	Negative	Negative	Negative	*NDM-1*						*CTX-M-15*	*TEM-181*		*OXA-1*		Yes	Yes (ompK36)
Kp 267	Positive	*K. pneumoniae*	ST395	2	O1/O2v1	Positive	Negative	Positive	bla_NDM_	Negative	Negative	Negative	Negative	Negative	*NDM-1*			*SHV-152*			*CTX-M-15*	*TEM-181*		*OXA-1*		*Yes*	Yes (ompK35)
Kp 300	Positive	*K. pneumoniae*	ST395	2	O1/O2v1	Positive	Negative	Positive	bla_NDM_	bla_VIM_	Negative	Negative	Negative	Negative	*NDM-1*						*CTX-M-15*	*TEM-181*		*OXA-1*		Yes	Yes (ompK36)
Kp 234	Positive	*K. pneumoniae*	ST17	25	O5	Positive	Positive	Negative	Negative	bla_VIM_	Negative	bla_KPC_	Negative	Negative											*OXA-9*	*No*	Yes (ompK37)
Kp 241	Positive	*K. pneumoniae*	ST17	25	O5	Negative	Positive	Negative	Negative	Negative	Negative	bla_KPC_	Negative	Negative		*KPC-3*	SHV-61	SHV-152	*SHV-187*						*OXA-9*	*No*	No
Kp 325	Positive	*K. pneumoniae*	ST17	25	O5	Negative	Positive	Negative	Negative	Negative	Negative	bla_KPC_	Negative	Negative		*KPC-3*		*SHV-152*				*TEM-181*				*Yes*	Yes (ompK36)
Kp 324	Positive	*K. pneumoniae*	ST17	25	O5	Positive	Positive	Negative	Negative	bla_VIM_	Negative	bla_KPC_	Negative	Negative		*KPC-3*						*TEM-181*	*TEM-122*		*OXA-9*	*No*	Yes (ompK37)
Kp 323	Positive	*K. pneumoniae*	ST17	25	O5	Negative	Positive	Negative	Negative	Negative	Negative	bla_KPC_	Negative	Negative		*KPC-3*			*SHV-187*			*TEM-181*			*OXA-9*	*No*	Yes (ompK36)
Kp 209	Positive	*K. pneumoniae*	ST101	17	O1/O2v1	Negative	Positive	Negative	Negative	Negative	Negative	bla_KPC_	Negative	Negative		*KPC-3*			*SHV-187*	*SHV-212*						*No*	Yes (ompK36, ompK37)
Kp 244	Positive	*K. pneumoniae*	ST101	17	O1/O2v1	Positive	Positive	Negative	bla_NDM_	Negative	Negative	bla_KPC_	Negative	Negative		*KPC-3*			*SHV-187*	*SHV-212*						*Yes*	Yes (ompK36, ompK37)
Kp 230	Positive	*K. pneumoniae*	ST101	17	O1/O2v1	Negative	Positive	Negative	Negative	Negative	Negative	bla_KPC_	Negative	Negative		*KPC-3*			*SHV-187*	*SHV-212*						*No*	Yes (ompK36)
Kp 312	Positive	*K. pneumoniae*	ST101	17	O1/O2v1	Negative	Positive	Positive	Negative	Negative	Negative	bla_KPC_	Negative	Negative		*KPC-3*						*TEM-181*			*OXA-9*	*No*	No
Kp 316	Positive	*K. pneumoniae*	ST307	102	O1/O2v2	Negative	Positive	Positive	Negative	Negative	Negative	bla_KPC_	Negative	Negative		*KPC-3*						*TEM-181*		*OXA-1*		*No*	No
Kp 341	Positive	*K. pneumoniae*	ST307	102	O1/O2v2	Negative	Positive	Negative	Negative	Negative	Negative	bla_KPC_	Negative	Negative		*KPC-3*					*CTX-M-15*		*TEM-122*	*OXA-1*	*OXA-9*	*No*	No
Kp 285	Positive	*K. pneumoniae*	ST307	102	O1/O2v2	Negative	Positive	Negative	Negative	Negative	Negative	bla_KPC_	Negative	Negative		*KPC-3*					*CTX-M-15*	*TEM-181*	*TEM-122*	*OXA-1*	*OXA-9*	*No*	No
Kp 327	Positive	*K. pneumoniae*	ST46	64	O1/O2v1	Negative	Positive	Negative	Negative	Negative	Negative	bla_KPC_	Negative	Negative		*KPC-3*										No	Yes (ompK37)
Kp 248	Positive	*K. pneumoniae*	ST46	64	O1/O2v1	Negative	Positive	Negative	Negative	Negative	Negative	bla_KPC_	Negative	Negative		*KPC-3*	*SHV-61*	*SHV-152*				*TEM-181*			*OXA-9*	*No*	Yes (ompK36, ompK37)
Kp 232	Positive	*K. pneumoniae*	ST35	22	O1/O2v1	Negative	Positive	Negative	Negative	Negative	Negative	bla_KPC_	Negative	Negative		*KPC-3*			*SHV-187*			*TEM-181*				*No*	Yes (ompK36, ompK37)

**Table 6:** Comparison between Carba-Screen results and NGS resistome analysis related to *A. baumannii* (Ab) isolates.

					NGS (resistome analysis related to β-lactam resistance)		
				Realquality carbascreen screening (1° level)	bla_OXA_		
Strain	Carbapenem-resistant phenotype	Identification	MLST	*Acinetobacter* OXA	OXA-51	OXA-23	OXA-66
Ab 292	Positive	*A. baumannii*	ST2	Positive	Positive	Positive	Negative
Ab 230/2	Positive	*A. baumannii*	ST2	Positive	Positive	Positive	Negative
Ab 250	Positive	*A. baumannii*	ST2	Positive	Positive	Positive	Positive
Ab 311	Positive	*A. baumannii*	ST2	Positive	Positive	Positive	Positive

## 4 Discussion

Surveillance protocols represent an essential resource in facing and containing the antimicrobial resistance increase. According to surveillance networks e recent literary data, rectal swabs may be an optimal candidate to detect multi-drug microorganisms as initial gut commensals (Adler et al., 2011; Tacconelli et al., 2014; Lee et al., 2015; Foschi et al., 2020; Fasciana, 2023). The molecular technologies may ensure a resistance markers detection directly from rectal swabs, demonstrating convincing agreement rates with conventional culture-based methods (Lerner et al., 2013; Viale et al., 2015). Our experimental study aimed to evaluate the REALQUALITY Carba-Screen kit (AB ANALITICA, Padova, Italy) in detecting carbapenems and colistin resistance markers of *Enterobacterales* and *Acinetobacter* spp. The study compared its molecular performance to the traditional culture-based method. Furthermore, the additional next-generation sequencing technology further elaborated and confirmed the presence of resistant isolated and their markers, outlining an important epidemiological context.

The Carba-Screen molecular assay demonstrated optimal sensitivity rates, confirming the possibility of detecting resistance markers despite the occasional presence of low clinical material quantity. Additionally, a 100% negative predictive value revealed the high probability of rapidly excluding the presence of carbapenem-resistant microorganisms. A discrete number of culture-negative samples with a molecular positive result emerged from the study. This molecular positive result was confirmed after the application of the Cepheid GeneXpert Carba-R methodology on the same rectal swabs, whose cartridges are normally used to confirm resistance markers on grown colonies within our diagnostic routine. On one side, the presence of these culture-negative and molecular positive results slightly affected specificity, positive predictive value rates, and statistical agreement. On the other side, this aspect may be considered an advantage, highlighting the molecular assay’s capability to detect resistance markers within low-inoculum positive samples or previously broad-spectrum treated patients. Moreover, previously published studies about molecular technologies documented similar results, enhancing occasional low positive predictive values but high sensitivity and overall agreement rates (EUCAST, 2023). On that premise, the high sensitivity rate is the most important feature clinicians require to better manage colonized patients. A restricted percentage of the analyzed samples revealed an invalid result. We hypothesized a low bacterial count and further investigations by fluorometric assays (data not shown) demonstrated the hypothesis of a poor or absent microbial DNA quantification. Moreover, the culture exams related to these specific samples confirmed a totally negative result. No relationships between invalid results and specific clinical setting have been reported.

The Carba-Screen assay perfectly matched the phenotypical results about β-lactams MIC values of the corresponding isolated strains. The assay efficiency reflected the actual epidemiological condition (Regione siciliana, 2019; Ochońska et al., 2021; EARS-Net, 2023), which describes increasing antimicrobial resistance levels among specific European areas. Notably, the two-step procedure allows organizing a specific and prompt patient’ coorting, due to the rapid qualitative response about eventual positive results. Our experience really appreciated the possibility of detecting both *Acinetobacter* OXA (first level) and *mcr* genes (second level), which are uncommon targets for current molecular diagnostic technologies. The restricted time interval to produce a resistance marker result suggests the hypothesis to extend the surveillance protocols to all the possible inter-wards patients’ transfers. This option could lead to better infection control measures, enhancing antimicrobial resistance awareness within all the hospital setting units. In addition, fast surveillance procedures may contain overtreatment predicting actual resistance markers. Insufficient evidence supports the effectiveness of decontamination treatments in colonized patients, especially within health-care settings with a high antimicrobial resistance prevalence (Shaidullina et al., 2023). The possibility to noticeably exclude resistant microorganisms and encourage a prompt infection control procedure within critical wards reduce the indiscriminate antibiotics spread against colonizing strains. In our opinion, the rapidity and sensitivity of the molecular assay may be an interesting solution in containing overtreatment about non-symptomatic colonized patients, which only need isolation protocols and infection control measures application.

The NGS analysis highlighted interesting epidemiological and resistance data. First of all, *K. pneumoniae* strains belonged to really diversified ST, which appeared different from the clinically significative *K. pneumoniae* strains (FASTQ, 2021). Ten out of 26 isolates showed the ST395, including emerging *K. pneumoniae* carbapenem-resistant high-risk clones (Maida et al., 2018). The ST395 has been extensively described among European countries, but its presence within the Southern Italy regions is relatively recent (FASTQ, 2021). Moreover, literary data document an OXA-48 and ST395 frequent combination, while our results reported a strict association between the ST395 and NDM carbapenemases genes. Otherwise, our ST395 strains showed the recurring detection of SHV-187, supporting previous data which described the association between this sequence type and SHV alleles (De Koster et al., 2022).

Finally, all the reported ST395 *K. pneumoniae* strains showed the capsular type k2, which has already been recognized as an alert type due to the simultaneous presence of high-risk virulence and resistance markers (Hetland et al., 2023).

A number of 4 *K. pneumoniae* strains belonged to ST101, while three isolates showed the ST307. Both these STs incorporate carbapenem-resistance high-risk clones frequently described as systemic infections etiological agents. Five strains reported the ST17, which is already known for gastro-intestinal commensalism and intensive care unit persistence (Frenk et al., 2020).

Two strains belonged to ST46, already described as an extended β-lactamases (ESBL) and OXA producer (Marcade et al., 2013). One of our isolates only revealed KPC and porine gene alterations, while the other reported SHV, TEM, KPC and OXA genes together with a non-*mcr-1* related colistin resistance (MIC = 8 mg/L). These considerations will probably deserve further investigations in the future, with the aim to establish the actual colistin resistance mechanism.

One single strain belonged to the ST35, globally known for to its ubiquitous distribution. Literary data often associate the ST35 with ESBL genes such as SHV and CTX-M (Nawfal Dagher et al., 2019). According to this assumption, our strain reveals the SHV-187 and the less common TEM-181, which has not already described within this ST.

As regards *A. baumannii*, all the strains belonged to the ST2, described as the main European pandemic *A. baumannii* lineage. Most literary data report the ST2 as the crucial carbapenem-resistance OXA gene carrier. According to this evidence, our results perfectly match the global epidemiological condition (Osman et al., 2020; Rezaei et al., 2020; Fonseca et al., 2023). Two analyzed strains revealed the detection of OXA-66, described as an OXA-51 family member (Gupta et al., 2022).

The WGS analysis performed on culture-positive isolates (29 samples) not always matched the Carba-Screen detection (7 cases). Specifically, 6 cases reported a Carba-Screen double carbapenem-resistance markers (NDM and KPC) detection, while the NGS analysis confirmed only one marker (NDM or KPC). On the other hand, one strain revealed a Carba-Screen VIM and KPC detection, but the NGS analysis retracted both carbapenemases genes presence. However, the carbapenem-resistance phenotype was explained by the presence of the OXA-9 gene and porine gene alterations.

Certainly, our protocol suffers from some weaknesses. First, a short experimental period (1 month) has been documented. This disadvantage may lead to a restricted sample number and a limited circulating strains spectrum. Additionally, the intention to compare the molecular assay to the culture method affected the statistical evaluation, resulting in low specificity and predictive positive volumes rates. It would be interesting to extend the experimental workflow to longer time intervals, also comparing the REALQUALITY performance to other molecular technologies routinary used into the diagnostic workflow as previous authors described (EUCAST, 2023).

The fundamental purpose of our work is to strongly recommend the introduction of a fast molecular screening in extensive healthcare settings within the principal endemic areas for multi-drug resistant microorganisms. The efficacy of the molecular screening in terms of sensitivity, specificity and detection, has been extensively demonstrated through the application of a universal investigation method such as the next-generation sequencing. The possibility of a multi-step procedure is useful to rapidly provide preliminary results, resulting in coordinated infection control procedures which are subsequently confirmed by the molecular identification of precise resistance markers. The two-levels screening strategy is an added value comparing the analyzed kit to other molecular technologies. Indeed, the first level allows to exclude all the negative results from unuseful and expensive further laboratory procedures, also early alerting clinicians about positive samples. Some limitations of the two-levels procedures may be related to the need of a dedicated manual labor. Globally, the protocol could require a restricted time interval, ensuring a rapid patients’ coorting and an interesting sensitivity rate. Clinical settings such as inter-wards patients’ transfers or pre-surgical screening are ideal healthcare context to apply a molecular screening directly from biological samples.

The high sensitivity may occasionally identify resistance markers which are not confirmed by colonies growth on culture exams. In conclusion, the molecular screening could initially lead to a more conservative approach, which may be reevaluated after a definitive conventional result about the microorganisms’ identification and susceptibility profile.

## Data availability statement

The original contributions presented in the study are publicly available. This data can be found at: https://www.ncbi.nlm.nih.gov/bioproject/; PRJNA1063661.

## Ethics statement

The study was conducted according to the guidelines of the Declaration of Helsinki and the best clinical practice (D.M. 15/07/1997). The present study does not directly involve patient management or drug administration. The institutional document n.101/CECT2 approved the investigations on patients’ biological samples. The studies were conducted in accordance with the local legislation and institutional requirements. The ethics committee/institutional review board waived the requirement of written informed consent for participation from the participants or the participants’ legal guardians/next of kin because The study did not directly involve human beings or supplementary biological samples apart from the diagnostic routine.

## Author contributions

MC: Conceptualization, Data curation, Investigation, Methodology, Writing – original draft. GMi: Data curation, Investigation, Methodology, Writing – original draft. GMa: Data curation, Investigation, Methodology, Writing – original draft. DB: Conceptualization, Investigation, Methodology, Supervision, Writing – review & editing. CB: Data curation, Investigation, Methodology, Writing – original draft. EN: Data curation, Investigation, Methodology, Writing – original draft. GS: Supervision, Writing – review & editing. SS: Conceptualization, Supervision, Writing – review & editing.

## Footnotes

^1^ https://card.mcmaster.ca/home
